# Linking the triglyceride-glucose index to major adverse cardiocerebrovascular events in individuals with cardiovascular-kidney-metabolic syndrome: insights from an aggregate-data systematic review and dose–response meta-analysis

**DOI:** 10.3389/fendo.2026.1959631

**Published:** 2026-09-24

**Authors:** Leilei Liu, Tingting Yang, Linyuan Zhang, Jianqin Zhong, Zixuan Xu, Xiao Zhang, Jiyu Chen, Jieyi He, Kaya Xu, Yan He, Feng Hong

**Affiliations:** 1 Clinical Trials Center & Key Laboratory of Clinical Pharmacology and Translational Research, The Affiliated Hospital of Guizhou Medical University, Guiyang, China; 2 School of Public Health, the Key Laboratory of Environmental Pollution Monitoring and Disease Control, Ministry of Education, Guizhou Medical University, Guiyang, China; 3 Center for Diseases Control and Prevention of Yunyan District, Guiyang, China; 4 Department of Neurosurgery, The Affiliated Hospital of Guizhou Medical University, Guiyang, China

**Keywords:** cardiocerebrovascular diseases, CKM syndrome, meta-analysis, risk, triglyceride-glucose

## Abstract

**Background:**

Epidemiological research has indicated a potential association between the triglyceride-glucose (TyG) index and the risk of major adverse cardiocerebrovascular events (MACCE). Nevertheless, the precise nature and magnitude of this relationship remain to be fully elucidated.

**Objectives:**

This study aims to assess the dose–response relationship between the TyG index and MACCE among individuals with cardiovascular-kidney-metabolic (CKM) syndrome.

**Methods:**

A comprehensive literature search was conducted using the PubMed and Web of Science databases to identify relevant studies on the TyG-MACCE relationship up to 16 June 2026. Data were synthesized using both random- and fixed-effects models, and nonlinear dose–response meta-analyses were performed using restricted cubic spline models.

**Results:**

The analysis incorporated eight cohort studies, encompassing over two million participants and 179 thousand MACCE cases. The aggregated risk estimate for MACCE, comparing the highest to the lowest categories of TyG index exposure, was 1.20 (95% confidence interval [CI] *=* 1.11–1.30, *I*^2^ = 77.2%, *p*_heterogeneity_ < 0.001). The dose–response analysis revealed a nonlinear positive association between the TyG index and MACCE risk (*p*_nonlinear_ < 0.001), with summary risk estimates calculated for each 1-unit increment in the TyG index being 1.05 (95% CI *=* 1.02–1.08, *I*^2^ = 81.1%, *p*_heterogeneity_ < 0.001). Subgroup and sensitivity analyses yielded similar results.

**Conclusions:**

This suggests that the TyG index may contribute to adverse MACCE in individuals with CKM syndrome. Further studies are needed to confirm causality and the predictive power of the TyG index.

The research was prospectively registered in the Prospective Register for Systematic Reviews (registration number: CRD420261278535).

**Systematic Review Registration:**

https://www.crd.york.ac.uk/PROSPERO/view/CRD420261278535, identifier CRD420261278535.

## 1 Introduction

Modifying insulin resistance (IR) can influence the risk of major adverse cardiocerebrovascular events (MACCE) (1–3). The triglyceride-glucose (TyG) index has recently become a noninvasive, simple, and cost-effective indicator of IR, and is useful for predicting outcomes across various diseases (4, 5).

Cardiovascular-kidney-metabolic (CKM) syndrome (6, 7), an integrated pathophysiological framework encompassing cardiometabolic and cardiorenal diseases, affects over 80% of adults (8, 9), often progresses to advanced stages (10), and is associated with an increased disease burden among affected patients (11–13), underscoring the need for integrated strategies to prevent progression to advanced stages.

IR significantly impacts CKM syndrome development and outcomes, and existing comprehensive data from population-based studies show the TyG index’s role in predicting MACCE progression in patients with CKM syndrome (14–24). Data from multiple epidemiological studies have indicated an increased MACCE risk associated with elevated levels of the TyG index, suggesting its potential predictive value for adverse outcomes in CKM syndrome (15, 16, 18, 20). However, several studies have not supported a consistent TyG-MACCE relationship (17, 21, 23, 25), or have identified a positive association only at the highest levels of the TyG index (22), with some findings indicating merely a borderline risk in the highest (14) or third (19) quartile of the TyG index. Furthermore, recent analyses have demonstrated that the nonlinear dose–response relationship and the shape of the curve remain inconclusive and vary across studies (15, 16, 19, 20, 22–25). The relationship between the TyG index and MACCE also appears to be influenced by specific MACCE subtypes (24). Consequently, the prognostic utility of the TyG index for predicting MACCE in CKM syndrome has not been fully established.

The variability in findings across studies highlights the necessity for a more comprehensive understanding of the TyG-MACCE relationship and underscores the importance of identifying and characterizing potential biomarkers. Although several studies have explored these effects and provided preliminary insights, further investigation is warranted. In this context, we aim to synthesize the currently available evidence to quantify the TyG-MACCE dose–response relationship in individuals with CKM syndrome and address this clinical question through a comprehensive systematic review and meta-analysis.

## 2 Methods

### 2.1 Registration of protocol

The research was prospectively registered in the Prospective Register for Systematic Reviews (registration number: CRD420261278535). The study adhered to the Preferred Reporting Items for Systematic Reviews and Meta-Analyses guidelines (26) and the reporting guidelines for Meta-analyses of Observational Studies in Epidemiology (27).

### 2.2 Ethics approval

Since this study employed publicly accessible data, obtaining approval for the study protocol from an ethics committee or institutional review board was not required, nor was it necessary to obtain informed consent from study participants.

### 2.3 Data sources and search strategy

For the systematic search, PubMed and Web of Science were searched from their inception until 16 June 2026. The search was conducted without restrictions on language, country, or study design. Detailed search strategies can be found in **Supplementary Table S1**.

### 2.4 Study selection

Full-length articles were deemed eligible if they met the following criteria: (1) were conducted in adults with CKM syndrome; (2) involved exposure to the TyG index; (3) had an outcome of interest of MACCE, including incident cardiocerebrovascular diseases (CVDs) and deaths from CVDs, as well as subtypes of CVDs such as stroke, coronary heart disease (CHD), myocardial infarction, heart failure, etc.; and (4) reported the association between the TyG index and the study outcomes. In cases where the necessary data from the original article were unclear or unavailable and could not be obtained from other records within the same cohort, we attempted to contact the original authors via email to request additional information.

Furthermore, we excluded studies that were (1) reviews or meta-analyses; (2) letters to the editor; (3) focused on cumulative changes in the TyG index; (4) not conducted on the general population; or (5) contained unusable data. In cases of duplicate publications based on the same cohort, we included data from the most informative reports or those with the largest number of outcomes or the largest sample size.

After removing duplicates to identify relevant articles, the first author (Leilei Liu) and the second author (Tingting Yang) subsequently reviewed all titles and abstracts independently, as well as thoroughly reviewing full texts for articles deemed potentially eligible. If the authors did not agree on the information, a final decision was made through a majority vote involving a third author, Linyuan Zhang. The specific systematic search process is in **Supplementary Figure S1**.

### 2.5 Quality assessment

We independently assessed the quality of the studies using the 9-point Newcastle-Ottawa Scale (28). The following three domains were considered: (1) population selection, (2) intergroup comparability, and (3) outcome measurement (refer to **Supplementary Table S2**). To quantitatively assess the potential for reporting bias, we planned to employ Begg’s test (29) and Egger’s test.

### 2.6 Data extraction

For each eligible study, data were independently and repeatedly extracted by Leilei Liu and Tingting Yang, with any disagreements resolved through adjudication by a third author, Linyuan Zhang. The extracted data included study characteristics (such as the first author’s name, publication year, study name, country of origin, and study design), participant characteristics (including age, sex, sample size, and total cases), stages and definitions of CKM syndrome, outcomes, definitions of MACCE, categories of the TyG index, the numbers of participants and cases in each category of the TyG index, multivariable-adjusted hazard ratios (HRs) with 95% confidence intervals (CIs), and covariates in the fully adjusted model. This key information is summarized in **Supplementary Table S3**. To examine the dose–response relationship, pertinent studies were identified that reported continuous exposure metrics, such as risk estimates for MACCE per 1-unit increase in the TyG index, or compared multiple groups with at least three exposure categories.

### 2.7 Statistical analysis

We conducted a two-stage, random-effects meta-analysis of the dose–response model using Stata software (Version 12.1, Stata Corp, College Station, TX, USA). All *p-*values were two-sided, with a significance threshold set at 0.05. Initially, we assumed a linear association and calculated relative risks (RRs) and their corresponding 95% confidence intervals (CIs) for the MACCE risk per 1-unit increase in the TyG index, when sufficient data were available. The term RRs was employed as a generic descriptor for HRs (30). Subsequently, we aggregated the study-specific HRs to derive pooled RRs and their 95% CIs using the DerSimonian and Laird random-effects model (31). A fixed-effects model was applied when the *I*^2^ statistic was below 50%; otherwise, a random-effects model was utilized. For each study, the median or mean TyG index for each category was assigned to the corresponding risk estimate. In instances where the original article did not specify the median or mean TyG index per category, we used the midpoint of the upper and lower boundaries of each category as the mean TyG index. Furthermore, we assumed the interval width to be equivalent to that of the nearest category based on the open-ended highest or lowest category (32). When the number of MACCE cases or the number of participants for each exposure category was not specified, but the total number and the reported effect size were available, we utilized these values for estimation (33). Furthermore, to demonstrate the potential nonlinear dose–response relationship, we employed generalized least-squares regression with restricted cubic splines, placing knots at the 25th, 50th, and 75th percentiles of the exposure distribution, provided that more than two studies addressed the outcome of interest (34, 35). This was accompanied by a visualization of the results. The *p-*value for nonlinearity (*p*_nonlinear_) was calculated by testing the null hypothesis that the coefficient of the second spline is equal to zero (35, 36).

Heterogeneity among the studies was evaluated using the Cochran *Q* test and the *I*^2^ statistic (37). *I*^2^ values of approximately 25%, 50%, and 75% are interpreted as indicating low, moderate, and high heterogeneity, respectively (37, 38). In addition to the primary analysis, we performed a series of stratified analyses based on study location, sample size, number of MACCE cases, definition of MACCE, and adjustment for confounding variables. To evaluate the robustness of the synthesized results, we conducted the following sensitivity analyses: (1) to assess the impact of individual studies on the overall associations, we performed additional analyses by removing one study at a time using leave-one-out analysis; (2) repeated the meta-analysis using both random- and fixed-effects models (39); (3) excluded studies where the mean age of the study population exceeded 60 years; (4) restricted the analysis to participants with CKM syndrome in stages 0–3; (5) excluded studies with a median or average follow-up of less than 5 years; and (6) varied the number and position of the knots (10th, 30th, 70th, and 90th percentiles) based on previous studies to test the stability of the spline curve shape (40).

## 3 Results

### 3.1 Search results and characteristics of the included studies

Out of the 181 articles initially identified through duplicate removal, title and abstract screening, and full-text screening, eight cohort studies (15, 16, 20, 24, 25, 41–43) were selected for inclusion. The comprehensive literature search process is detailed in **Supplementary Figure S1**.

These studies, characterized by an observational cohort design, collectively involved 2,305,009 participants and reported 179,881 MACCE cases. The number of participants ranged from 6,867 (41) to 1,497,913 (16). Among these eight eligible studies (15, 16, 20, 24, 25, 41–43), six were conducted in Asia (15, 16, 20, 25, 41, 42), one in the USA (43), and one in the UK (24). Detailed data and statistics for each study are available in **Supplementary Table S2**.

According to the 9-point Newcastle-Ottawa Scale, all included studies were of high quality, with three studies (20, 42, 43) receiving a score of 9 and five studies (15, 16, 24, 25, 41) receiving a score of 8. The scores for each criterion of the NOS for each study are provided in **Supplementary Table S3**.

### 3.2 The highest versus lowest meta-analysis on the TyG-MACCE relationship in people with CKM syndrome

As illustrated in **Figure 1**, a meta-analysis of seven studies (15, 16, 20, 24, 25, 41, 43) revealed that individuals in the highest categories of the TyG index exhibited a 20% increased risk of MACCE compared with those in the lowest categories. The pooled RR was calculated to be 1.20, with a 95% CI of 1.11 to 1.30, and substantial heterogeneity was observed (*I*^2^ = 77.2%, *p*_heterogeneity_ < 0.001). Regarding other outcomes, including new-onset CVDs, cardiovascular mortality, incident stroke, and incident CHD, the highest TyG index categories also demonstrated statistically significant or borderline increases in risk compared with the lowest categories. The pooled RRs were 1.24 (95% CI = 1.15–1.34, *I*^2^ = 71.3%, *p*_heterogeneity_ = 0.008), 1.04 (95% CI = 0.94–1.15, *I*^2^ = 5.7%, *p*_heterogeneity_ = 0.303), 1.13 (95% CI = 0.97–1.32, *I*^2^ = 90.3%, *p*_heterogeneity_ < 0.001), and 1.29 (95% CI = 0.99–1.67, *I*^2^ = 99.2%, *p*_heterogeneity_ < 0.001), respectively, as depicted in **Supplementary Figures S2–S5**.

**Figure 1:**
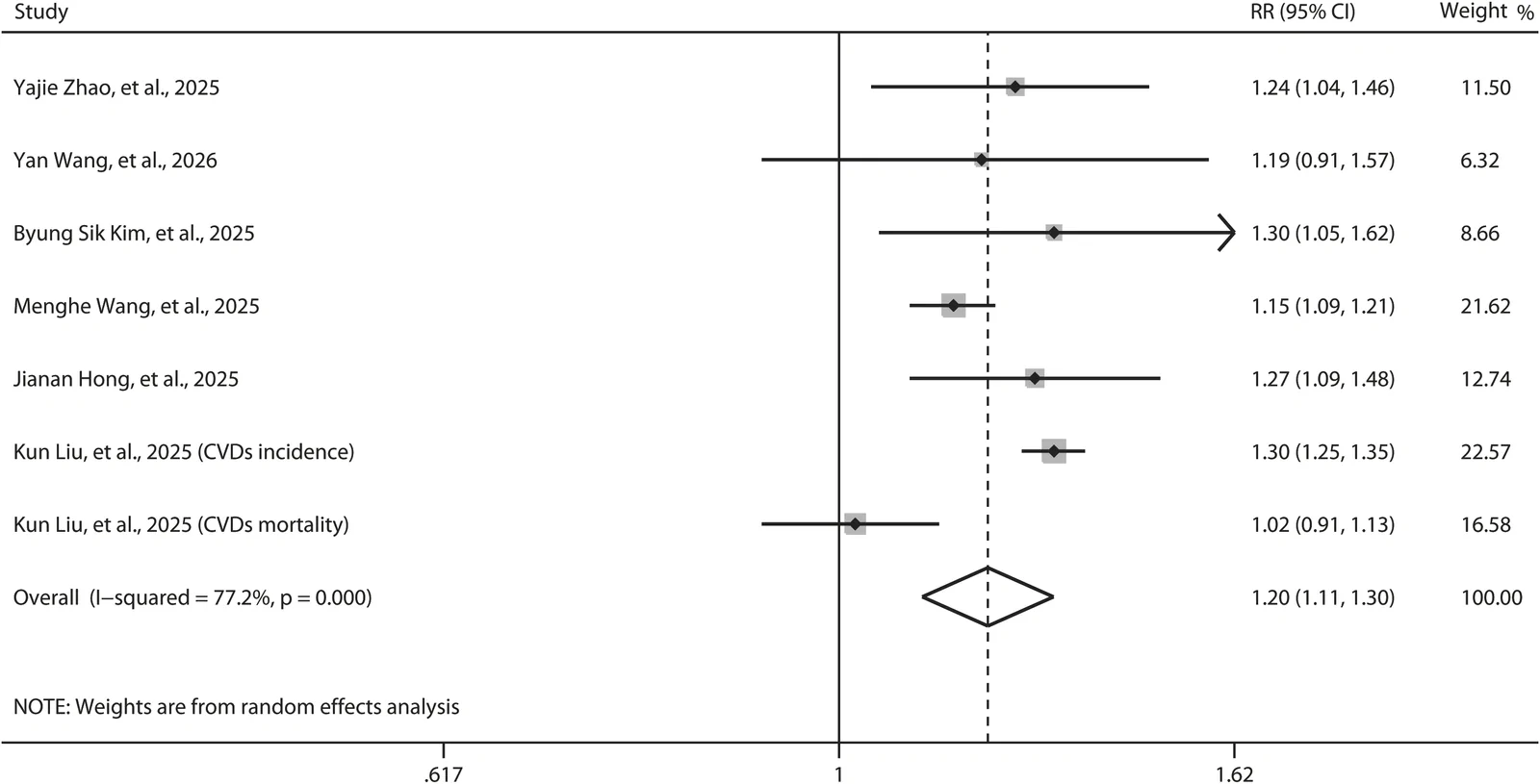
Summary of the relative risk of major adverse cardiac-cerebrovascular events associated with the highest versus lowest categories of the triglyceride-glucose index among individuals with cardiovascular-kidney-metabolic syndrome. CI, confidence interval; RR, relative risk.

No significant or credible differences were detected across the defined subgroups (**Supplementary Tables S4, S5**). The leave-one-out sensitivity analysis indicated consistent findings after excluding individual studies. Furthermore, similar associations between the TyG index and MACCE were observed in additional sensitivity analyses (**Supplementary Tables S6, S7**). Begg’s test and Egger’s test were nonsignificant (all *p* > 0.05), indicating no evidence of publication bias.

### 3.3 Dose–response analysis of the TyG-MACCE relationship in people with CKM syndrome

In summary, six studies (15, 16, 24, 25, 42, 43) were incorporated into a dose–response meta-analysis. As illustrated in **Figure 2**, each one-unit increase in the TyG index was correlated with a 5% elevation in the MACCE risk (pooled RR = 1.05, 95% CI = 1.02–1.08), with significant heterogeneity observed (*I*^2^ = 81.1%, *p*_heterogeneity_ < 0.001). Analogous results were noted for MACCE subtypes in relation to the TyG index dosage (**Supplementary Figures S6–S9**), including a 7% increased risk of incident CVDs (pooled RR = 1.07, 95% CI = 1.03–1.11, *I*^2^ = 69.4%, *p*_heterogeneity_ = 0.020), a 2% increased risk of incident stroke (pooled RR = 1.02, 95% CI = 0.99–1.05, *I*^2^ = 96.3%, *p*_heterogeneity_ = 0.020), and a 5% increased risk of incident CHD (pooled RR = 1.05, 95% CI = 1.00–1.11, *I*^2^ = 99.3%, *p*_heterogeneity_ < 0.001) associated with each 1-unit increase in the TyG index. Consistent findings emerged from subgroup and sensitivity analyses (**Supplementary Table S4–S7**). A leave-one-out analysis for MACCE risk revealed no significant alterations upon the exclusion of any single study. Furthermore, no evidence of publication bias was identified through Begg’s and Egger’s test analyses (all *p* > 0.05).

**Figure 2:**
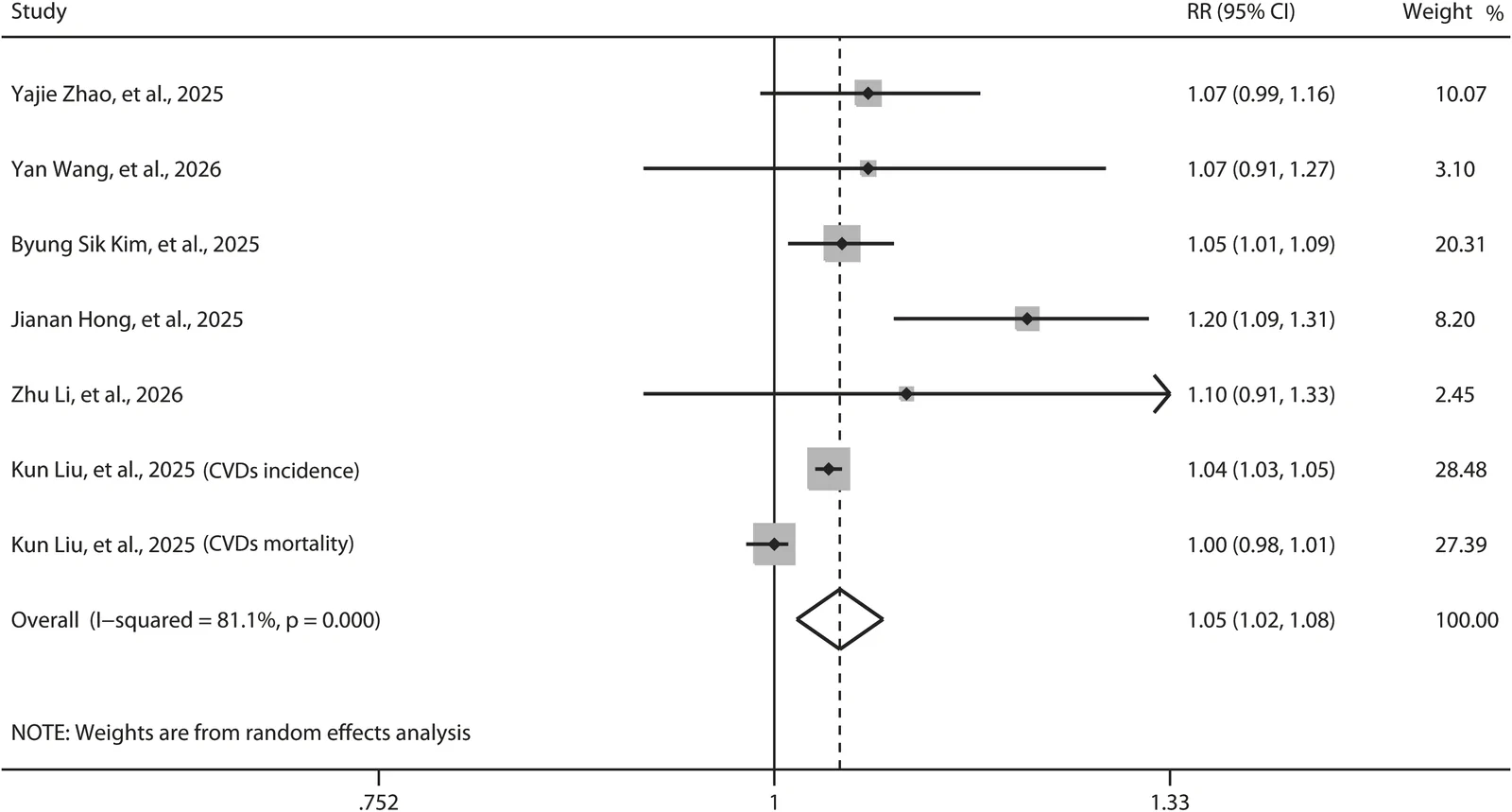
Forest plot of the study-specific relative risk of major adverse cardiac-cerebrovascular events per 1-unit increase in the triglyceride-glucose index in people with cardiovascular-kidney-metabolic syndrome. CI, confidence interval; RR, relative risk.

As depicted in **Figure 3**, the analysis indicated a nonlinear positive dose–response relationship, characterized by an increase in RRs with increasing levels of the TyG index (*p*_nonlinear_ < 0.001). Due to the limited number of studies, subgroup analysis was not conducted. Furthermore, the results of the additional sensitivity analyses were consistent with those of the primary analysis; detailed findings are provided in **Supplementary Figure S10**.

**Figure 3:**
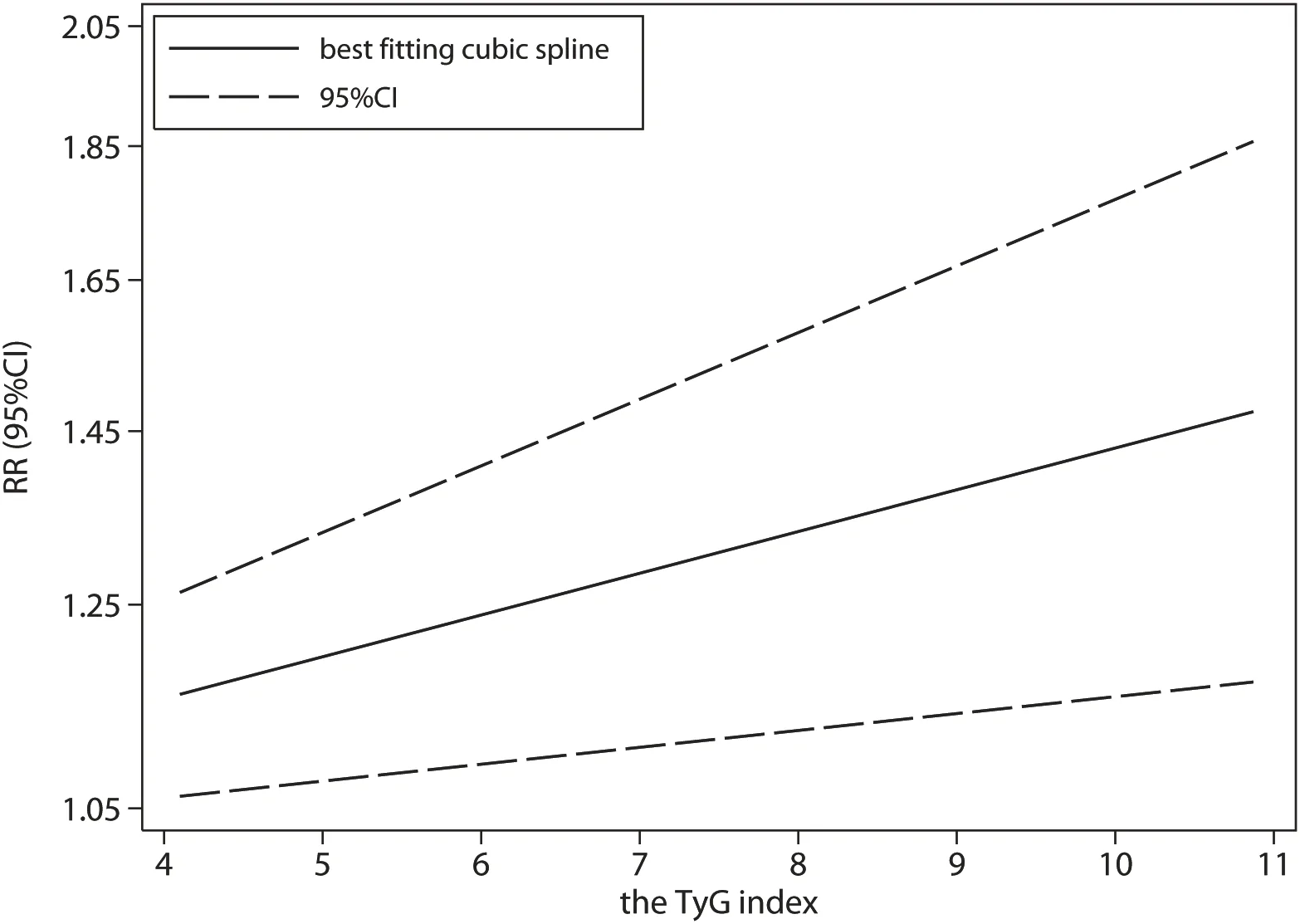
Dose–response association between the triglyceride-glucose index and the risk of major adverse cardiac-cerebrovascular events among participants with cardiovascular-kidney-metabolic syndrome modeled with restricted cubic splines. CI, confidence interval; RR, relative risk; TyG, triglyceride-glucose.

## 4 Discussion

To the best of our knowledge, this study represents the first dose–response meta-analysis synthesizing evidence on the relationship between the TyG index and the risk of MACCE among individuals with CKM syndrome. Our investigation, which included eight original publications encompassing 2,305,009 participants and 179,881 MACCE cases, suggests that elevated levels of the TyG index may increase the MACCE risk, as well as the incidence of CVDs and CVD-related mortality, in individuals with CKM syndrome.

Although the hyperinsulinemic–euglycemic clamp is considered the gold standard for assessing IR (44), its invasive, costly, and complex nature limits its applicability in large-scale epidemiological studies and clinical settings with constrained resources. Investigating suitable biomarkers for assessing the risk of MACCE in individuals with CKM syndrome is crucial for facilitating timely prevention, treatment, and risk stratification, as well as for predicting clinical outcomes. The TyG index, a composite measure derived from fasting triglyceride and plasma glucose levels, is cost-effective and widely accessible, and may serve as a valuable prognostic marker in CKM syndrome (4, 5, 45). The present findings underscore the importance of integrating both lipid metabolism and glycemic control into the assessment and early intervention of progressive cardiocerebrovascular risk. Furthermore, recent evidence regarding CETP-targeted strategies should be considered (46). Previous studies have demonstrated that the TyG index possesses superior predictive power for MACCE risk compared with other IR-related indices, such as the TyG-waist circumference, TyG-waist height ratio, TyG index with body mass index, the triglyceride-to-high-density lipoprotein cholesterol ratio, and the metabolic score for IR (47, 48), as well as the homeostasis model assessment of IR index, which evaluates *β*-cell function and IR (47, 49, 50). Based on these observations, we hypothesize that incorporating the TyG index, as a surrogate marker and biomarker for IR, into an established model for assessing MACCE risk in individuals with CKM syndrome may yield significant benefits.

TyG is one component of a broader cardiometabolic risk profile. Several potential molecular mechanisms, including imbalances in glucose metabolism, subsequent endothelial dysfunction, coagulation disorders, smooth muscle cell dysfunction, chronic low-grade inflammation, activation of the sympathetic nervous system, reduced vagal activity, activation of the renin-angiotensin-aldosterone system, renal sodium retention, and impaired cardiac calcium processing capacity, may potentially influence this relationship and partially explain the observed association (45, 51–58). The TyG index should be considered within the broader context of lipid transport (46), atherogenic lipoproteins, oxidative stress (59), diet, and lifestyle. However, the precise mechanisms underlying the TyG-MACCE relationship remain unclear.

Notably, current evidence regarding the predictive accuracy of the TyG index in assessing MACCE risk is limited. Despite this, some early observational studies indicated a nonsignificant (17, 21, 23) or borderline (14) TyG-MACCE association, as well as a positive relationship only in the high-dose groups (19, 22). These findings contrast with numerous studies utilizing population-based data that have not supported these findings (15, 16, 18, 20, 21, 25). Furthermore, the stress hyperglycemia ratio, an innovative metric for quantifying blood glucose fluctuations in patients experiencing acute or subacute stress, has shown a significant association with adverse cardiovascular outcomes and may influence the predictive value of the TyG index. Additionally, the TyG index may not be applicable to patients with hypertriglyceridemia and glucose metabolic disorders, underscoring the importance of dynamically monitoring longitudinal changes in the TyG index (60, 61). Moreover, it remains unclear which of the various surrogate markers for IR can best enhance the predictive performance of risk models for MACCE (47, 48, 62, 63). These conflicting data and uncertainties highlight substantial knowledge gaps regarding the application of the TyG index, which require urgent investigation and further validation.

Despite the strengths of this study, including the comprehensiveness of the search strategies and the extensive sensitivity analyses, which provide a thorough and quantitative summary of the TyG-MACCE relationship in individuals with CKM syndrome, several limitations warrant consideration. Firstly, MACCE represents a series of dynamic and progressive disturbances that occur irrespective of temporal changes in the TyG index, potentially resulting in a significant disease burden. However, to the best of our knowledge, there is a paucity of data on the relationship between cumulative changes in the TyG index and the risk of MACCE in individuals with CKM syndrome, with the exception of findings from the China Health and Retirement Longitudinal Study (14, 60). Future research should prioritize evaluating the mean changes in the TyG index in relation to MACCE progression during follow-up. Secondly, the limited number of stratified analyses in primary studies examining the dose–response relationship precluded the exploration of potential subgroup differences. Thirdly, due to the limited number of primary analyses, with the exception of data from the Korean National Health Insurance Database (16) and the UK Biobank (24), various forms of CVDs, such as stroke and CHD, were not thoroughly examined. Fourthly, although analyses of CVD incidence and mortality have been conducted, the small number of original studies investigating these relationships and the absence of data in most studies diminish the statistical power. Fifthly, given the high degree of heterogeneity observed, it is imperative to further investigate its sources. Furthermore, obtaining detailed individual-level data would enhance the robustness of the evidence presented in this study. Additionally, although the majority of studies included in this analysis were prospective in nature, acquiring interventional data is crucial to further strengthen the evidence base. The possibility of reverse causation should also be considered, given the observational design of the studies. Moreover, while most studies accounted for numerous associated confounding factors and data were extracted from the most comprehensively adjusted models, there was variability in how covariates were managed across studies. This introduces the possibility that residual or unknown confounders may have influenced our results, necessitating a cautious interpretation of the present findings. Additionally, due to the small number of included studies, publication bias could not be reliably assessed in this meta-analysis. Our study directly combined the RRs from eligible studies that employed different definitions for MACCE, as seen in some meta-analyses (64–66), potentially increasing the uncertainty of the estimates due to limited comparability. These limitations underscore the need for further evaluation in future research and should be taken into account when interpreting the results.

## 5 Conclusions

The TyG index may serve as an alternative surrogate marker for IR, potentially predicting the risk of MACCE among individuals with CKM syndrome. The dynamic monitoring and management of lipid and glucose metabolism are pivotal in facilitating the prevention and control of cardio-cerebrovascular risks within populations affected by CKM syndrome, ultimately enhancing overall well-being in adults. Future research employing high-quality and large-scale designs is necessary to establish a more detailed dose–response pattern and provide more comprehensive and robust evidence.

## Data Availability

The original contributions presented in the study are included in the article/**Supplementary Material**. Further inquiries can be directed to the corresponding authors.
