# Supplementary material for: Linking the triglyceride-glucose index to major adverse cardiocerebrovascular events in individuals with cardiovascular-kidney-metabolic syndrome: insights from an aggregate-data systematic review and dose–response meta-analysis
Source: Front Endocrinol (Lausanne). 2026 Sep 24;17:1959631. doi: 10.3389/fendo.2026.1959631 (PMC13645528; doi:10.3389/fendo.2026.1959631)
Supplement: Supplementary file 1 [file Image1.pdf]

Supplementary Figure legends

Supplementary Figure 1. Flow diagram of selection process for inclusion of studies in meta-analysis.

Supplementary Figure 2. Summary of relative risk for the risk of incident cardio-cerebrovascular diseases with the highest versus lowest categories of the triglyceride-glucose index among individuals with cardiovascular-kidney-metabolic syndrome. *CI*, *confidence interval*; *RR*, *relative risk.*

Supplementary Figure 3. Summary of relative risk for the risk of death from cardio-cerebrovascular diseases with the highest versus lowest categories of the triglyceride-glucose index among individuals with cardiovascular-kidney-metabolic syndrome. *CI*, *confidence interval*; *RR*, *relative risk.*

Supplementary Figure 4. Summary of relative risk for the risk of incident stroke with the highest versus lowest categories of the triglyceride-glucose index among individuals with cardiovascular-kidney-metabolic syndrome. *CI*, *confidence interval*; *RR*, *relative risk.*

Supplementary Figure 5. Summary of relative risk for the risk of incident coronary heart diseases with the highest versus lowest categories of the triglyceride-glucose index among individuals with cardiovascular-kidney-metabolic syndrome. *CI*, *confidence interval*; *RR*, *relative risk.*

Supplementary Figure 6. Forest plot of study-specific relative risk for the risk of incident cardio-cerebrovascular diseases for per one-unit increase in the triglyceride-glucose index in people with cardiovascular-kidney-metabolic syndrome.

CI, *confidence interval*; *RR*, *relative risk.*

Supplementary Figure 7. Forest plot of study-specific relative risk for the risk of death from cardio-cerebrovascular diseases for per one-unit increase in the triglyceride-glucose index in people with cardiovascular-kidney-metabolic syndrome. *CI*, *confidence interval*; *RR*, *relative risk.*

Supplementary Figure 8. Forest plot of study-specific relative risk for the risk of incident stroke for per one-unit increase in the triglyceride-glucose index in people with cardiovascular-kidney-metabolic syndrome. *CI*, *confidence interval*; *RR*, *relative risk.*

Supplementary Figure 9. Forest plot of study-specific relative risk for the risk of incident coronary heart diseases for per one-unit increase in the triglyceride-glucose index in people with cardiovascular-kidney-metabolic syndrome. *CI*, *confidence interval*; *RR*, *relative risk.*

Supplementary Figure 10. Sensitivity analyses by varing the numbers and position of the knots (10th, 30th, 70th, and 90th percentiles) for the dose-response association between the triglyceride-glucose index and the risk of major adverse cardio-cerebrovascular events among participants with cardiovascular-kidney-metabolic syndrome modeled by using restricted cubic splines. *CI*, *confidence interval*; *RR*, *relative risk;* TyG, triglyceride-glucose.

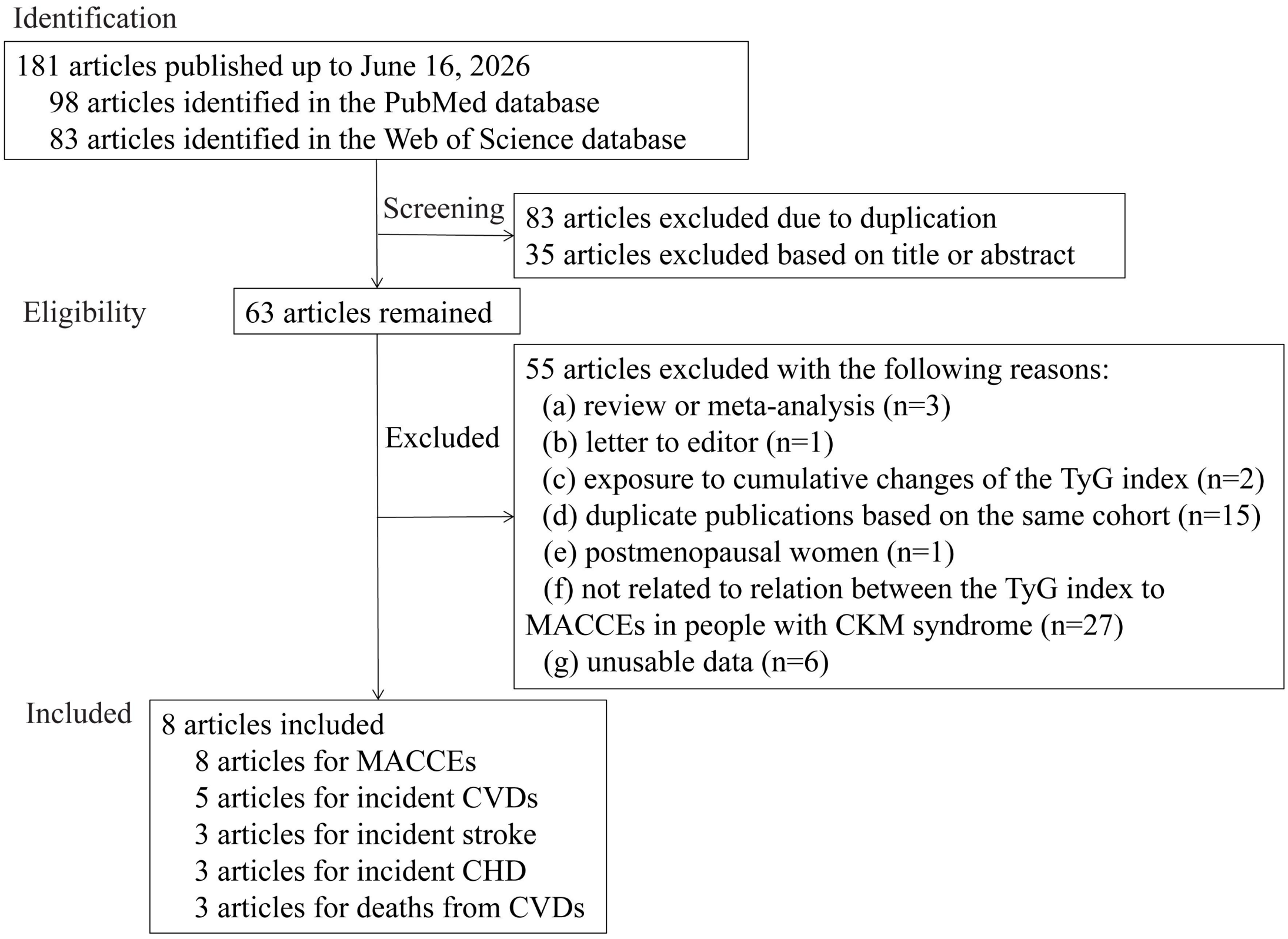

Supplementary Figure 1. Flow diagram of selection process for inclusion of studies in meta-analysis. CHD, coronary heart diseases; CKM, cardiovascular-kidney-metabolic; CVDs, cardio-cerebrovascular diseases; MACCEs, major adverse cardio-cerebrovascular events; TyG, triglyceride-glucose.

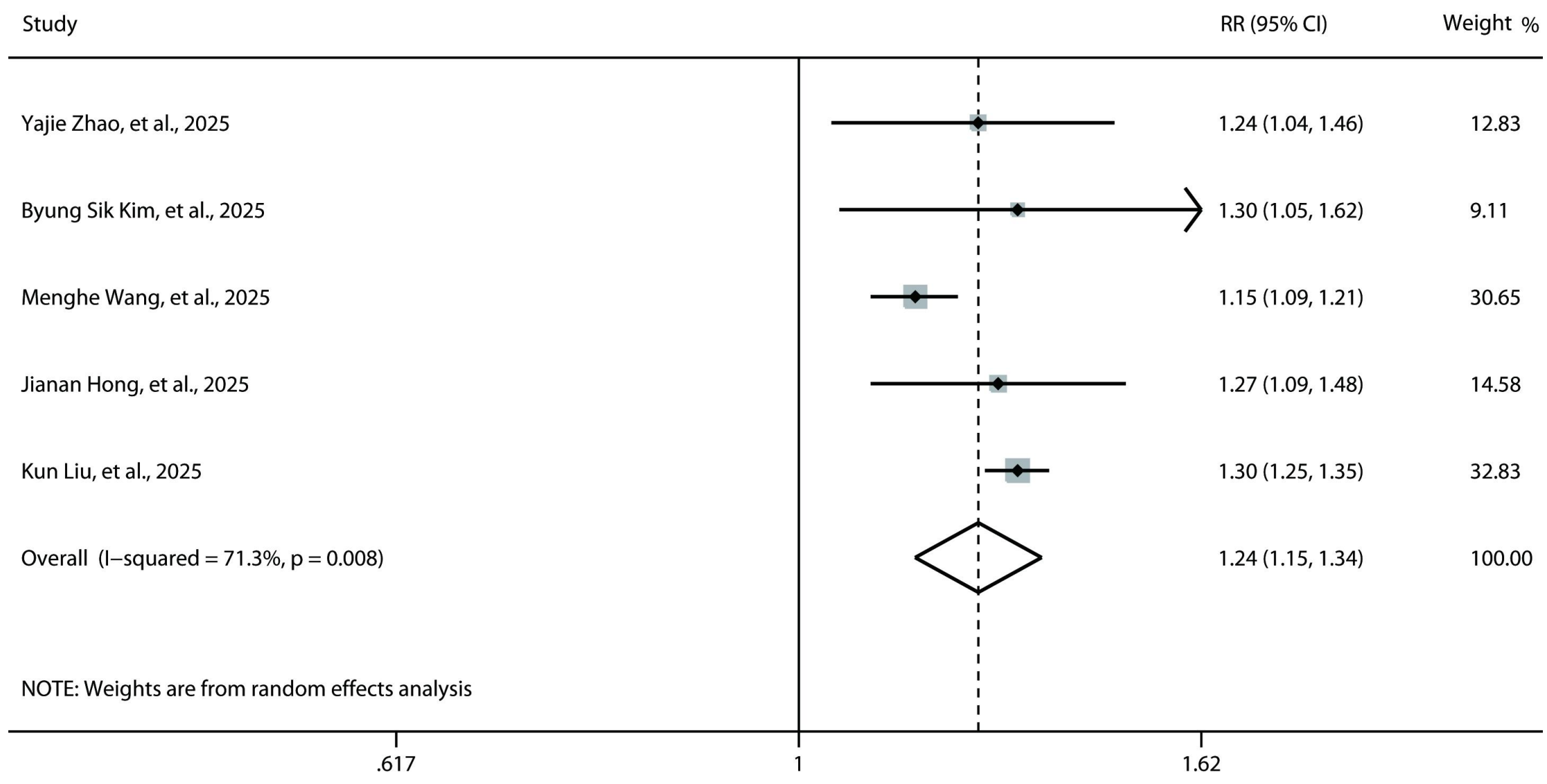

Supplementary Figure 2. Summary of relative risk for the risk of incident cardio-cerebrovascular diseases with the highest versus lowest categories of the triglyceride-glucose index among individuals with cardiovascular-kidney-metabolic syndrome. *CI*, *confidence interval*; *RR*, *relative risk.*

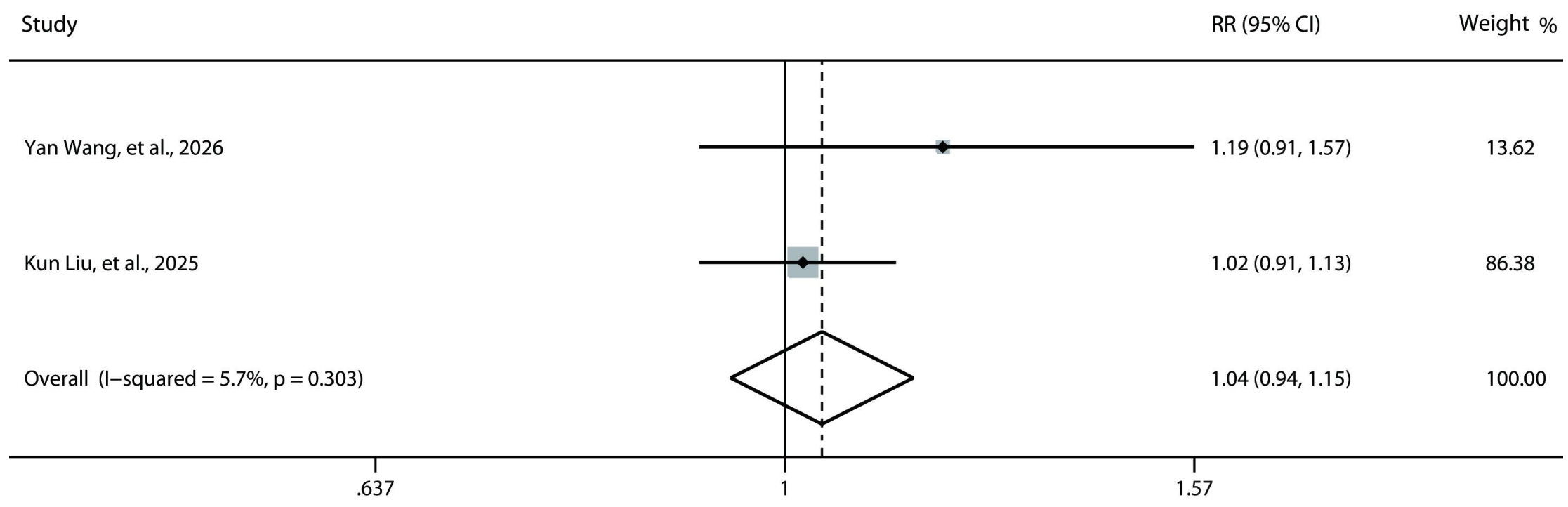

Supplementary Figure 3. Summary of relative risk for the risk of death from cardio-cerebrovascular diseases with the highest versus lowest categories of the triglyceride-glucose index among individuals with cardiovascular-kidney-metabolic syndrome. *CI*, *confidence interval*; *RR*, *relative risk.*

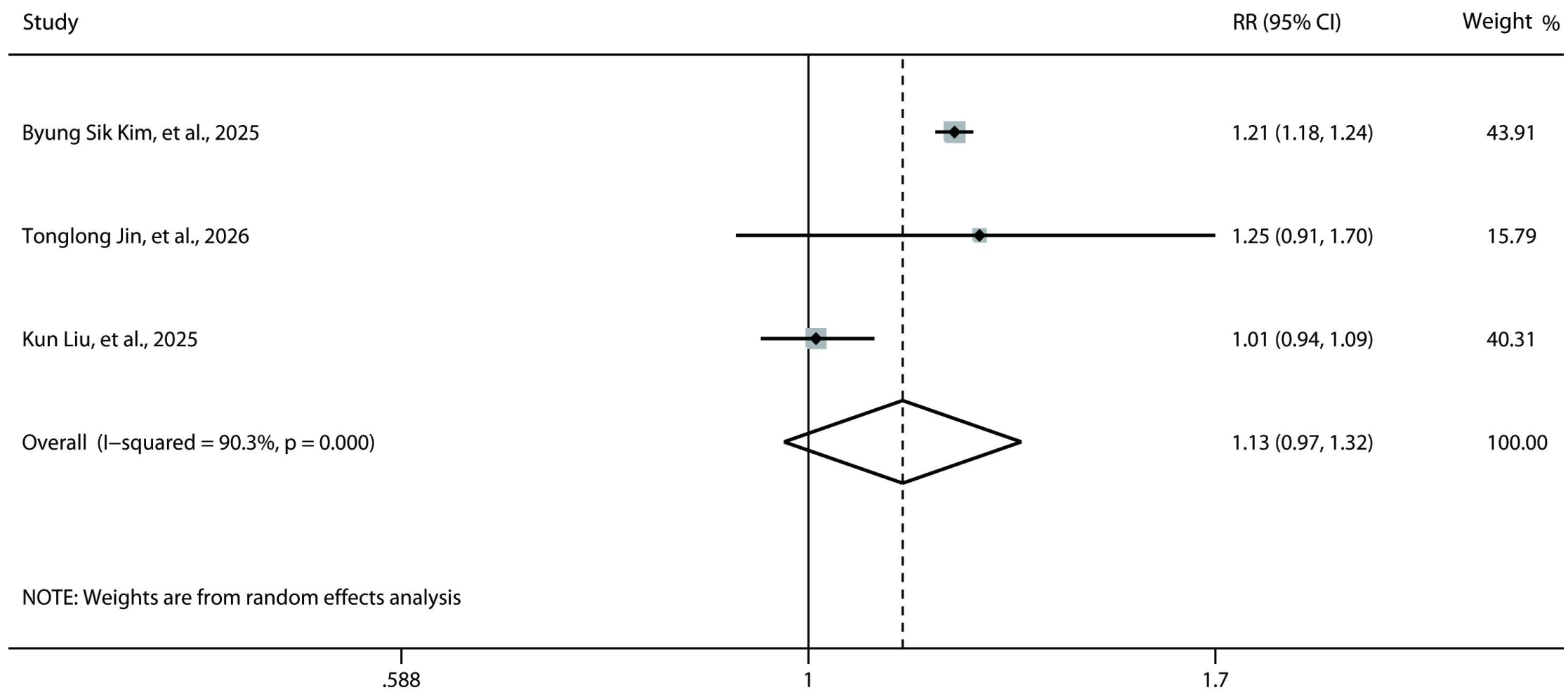

Supplementary Figure 4. Summary of relative risk for the risk of incident stroke with the highest versus lowest categories of the triglyceride-glucose index among individuals with cardiovascular-kidney-metabolic syndrome. *CI*, *confidence interval*; *RR*, *relative risk.*

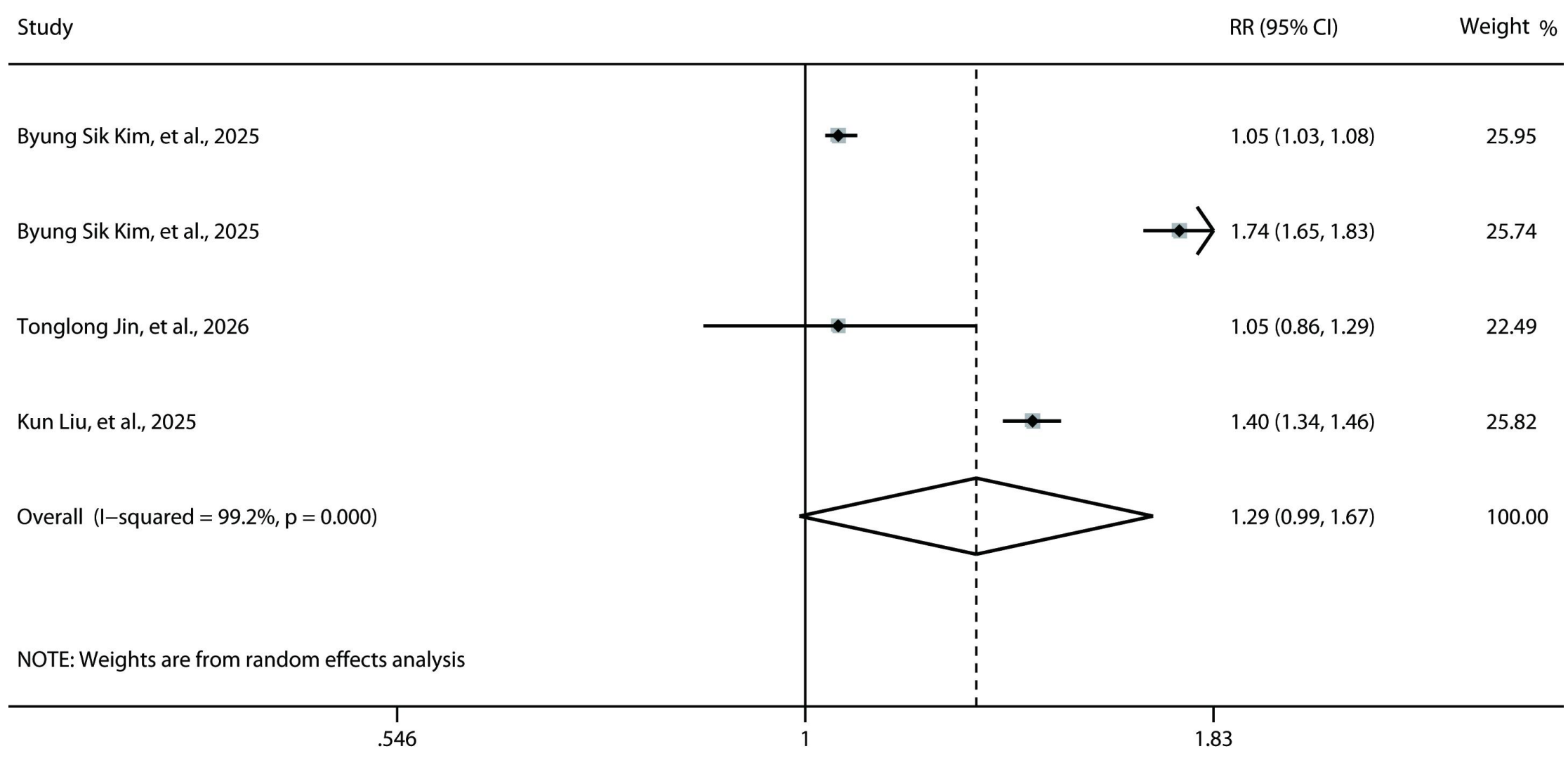

Supplementary Figure 5. Summary of relative risk for the risk of incident coronary heart diseases with the highest versus lowest categories of the triglyceride-glucose index among individuals with cardiovascular-kidney-metabolic syndrome. *CI*, *confidence interval*; *RR*, *relative risk.*

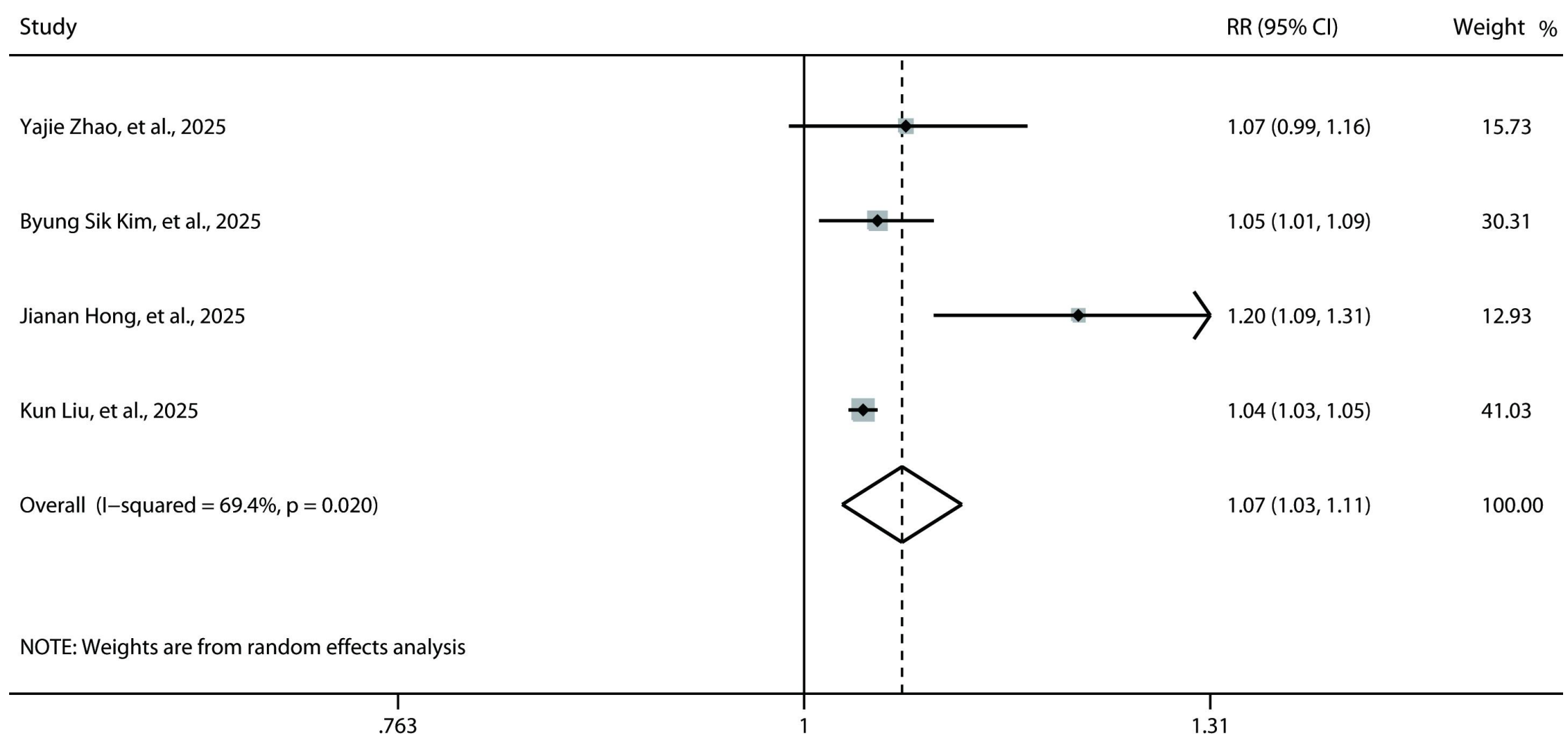

Supplementary Figure 6. Forest plot of study-specific relative risk for the risk of incident cardio-cerebrovascular diseases for per one-unit increase in the triglyceride-glucose index in people with cardiovascular-kidney-metabolic syndrome. *CI*, *confidence interval*; *RR*, *relative risk.*

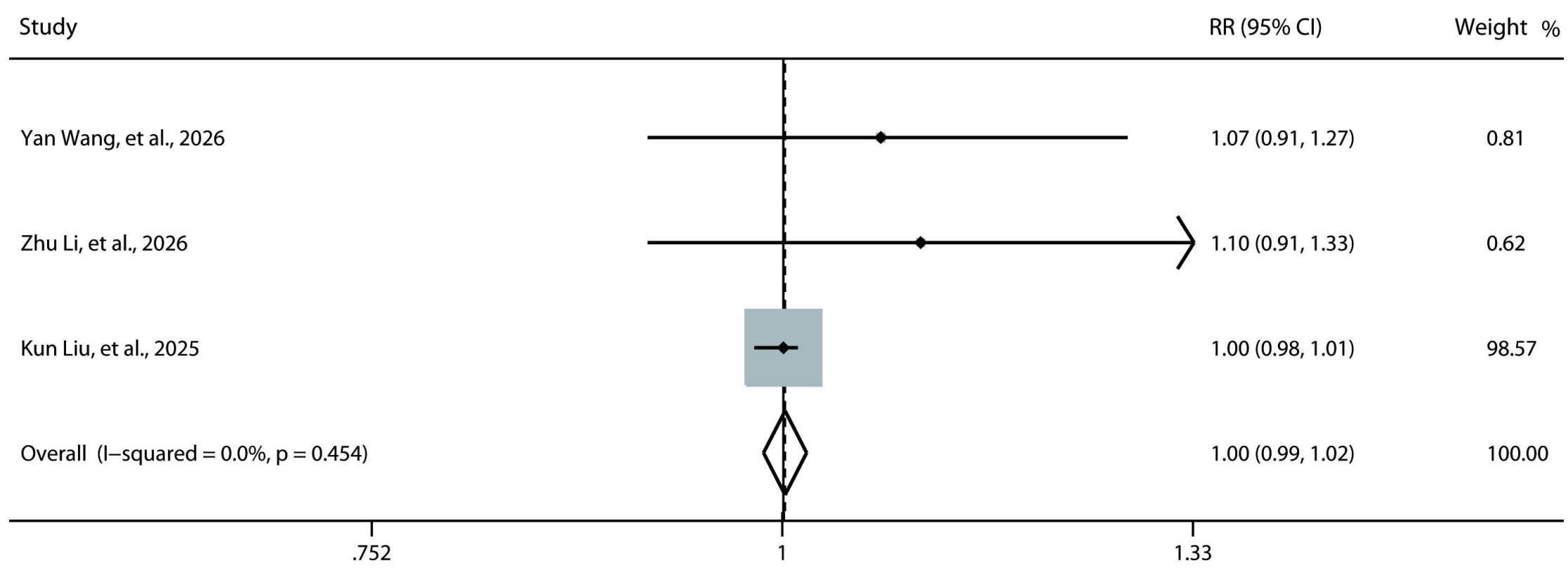

Supplementary Figure 7. Forest plot of study-specific relative risk for the risk of death from cardio-cerebrovascular diseases for per one-unit increase in the triglyceride-glucose index in people with cardiovascular-kidney-metabolic syndrome. *CI*, *confidence interval*; *RR*, *relative risk.*

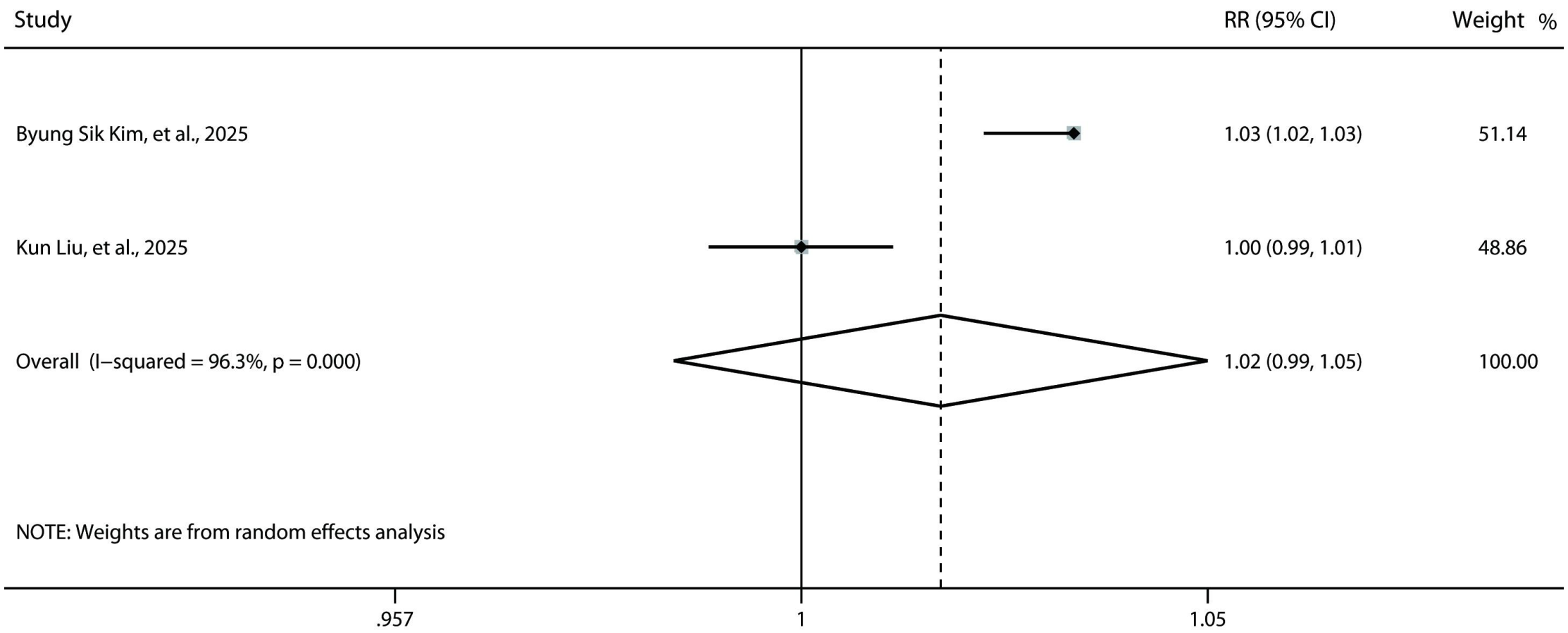

Supplementary Figure 8. Forest plot of study-specific relative risk for the risk of incident stroke for per one-unit increase in the triglyceride-glucose index in people with cardiovascular-kidney-metabolic syndrome. *CI*, *confidence interval*; *RR*, *relative risk.*

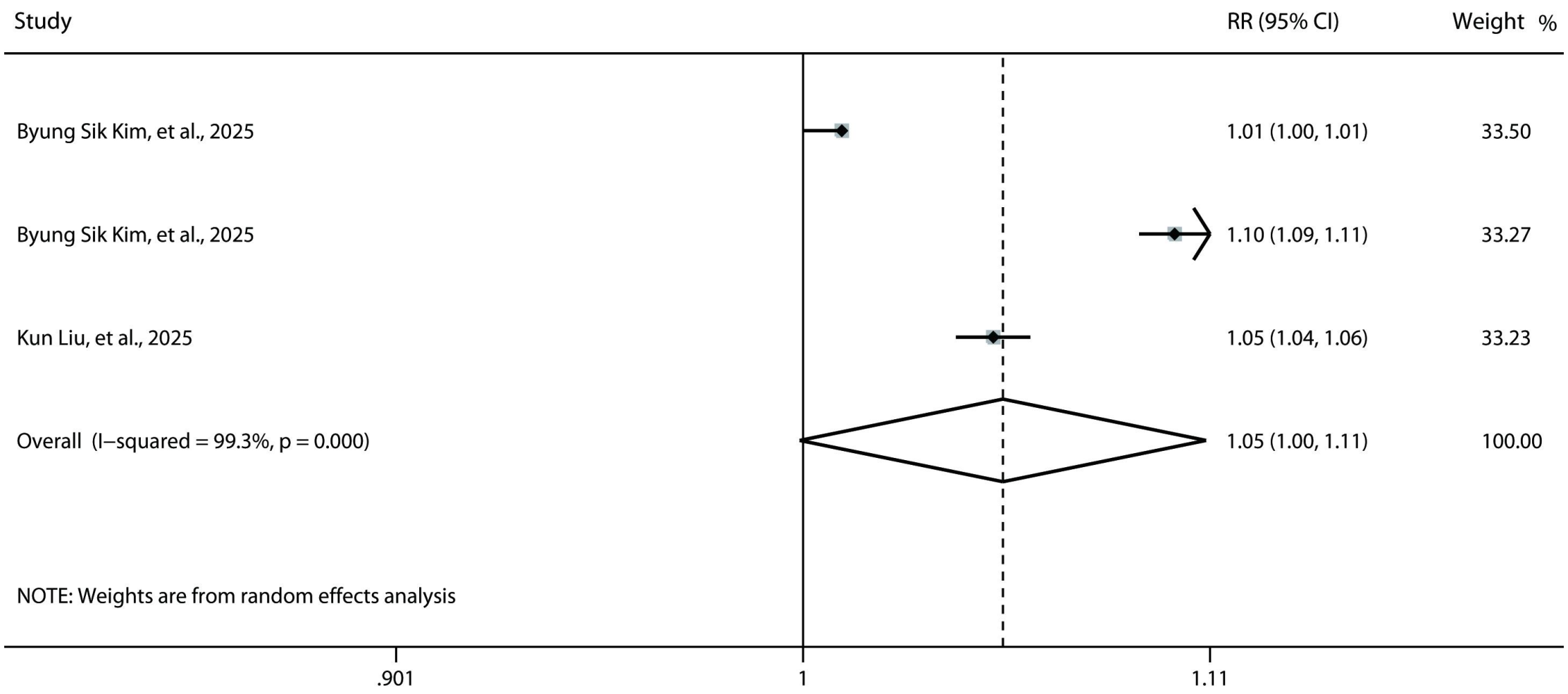

Supplementary Figure 9. Forest plot of study-specific relative risk for the risk of incident coronary heart diseases for per one-unit increase in the triglyceride-glucose index in people with cardiovascular-kidney-metabolic syndrome. *CI*, *confidence interval*; *RR*, *relative risk.*

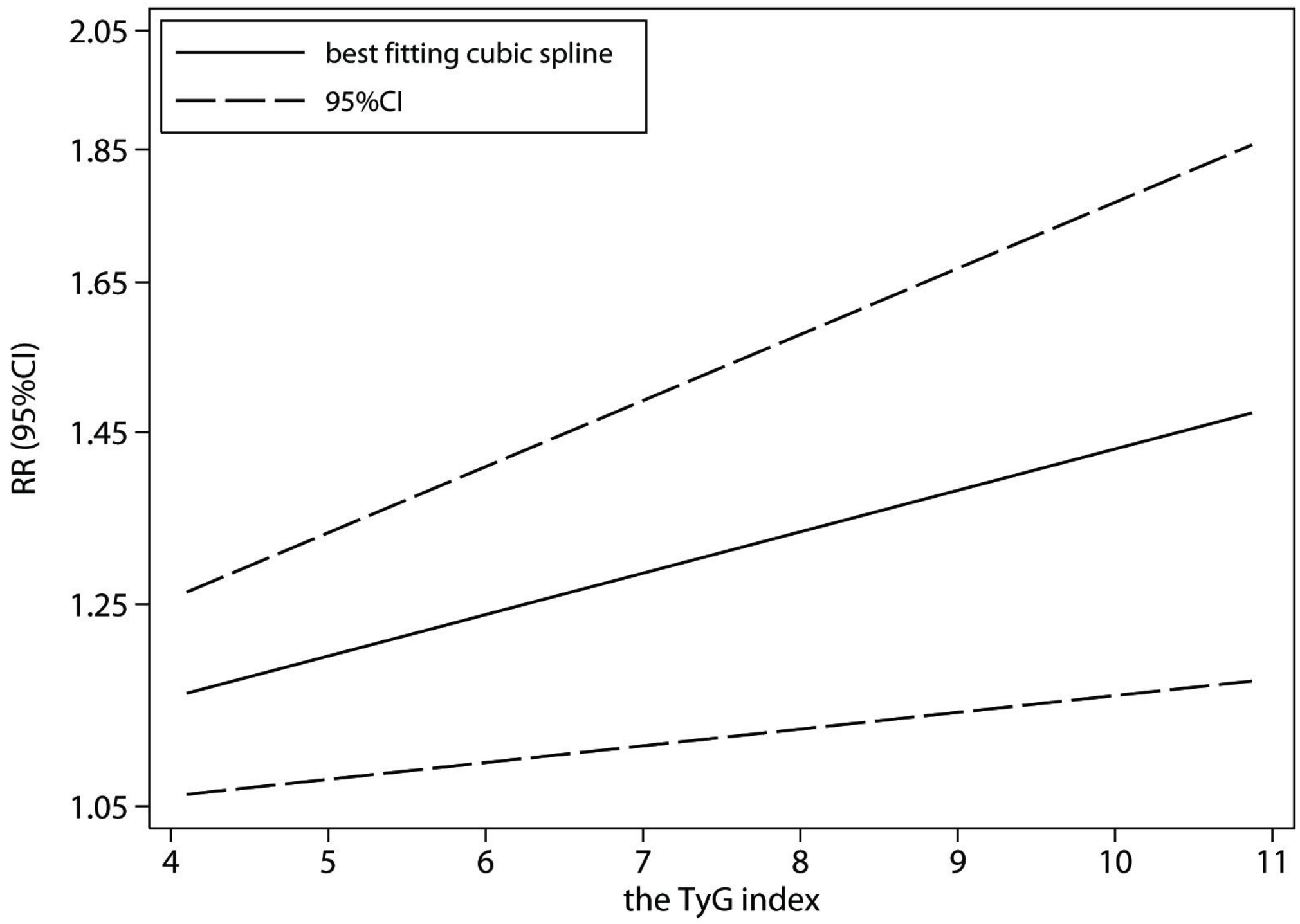

Supplementary Figure 10. Sensitivity analyses by varing the numbers and position of the knots (10th, 30th, 70th, and 90th percentiles) for the dose-response association between the triglyceride-glucose index and the risk of major adverse cardio-cerebrovascular events among participants with cardiovascular-kidney-metabolic syndrome modeled by using restricted cubic splines. *CI*, *confidence interval*; *RR*, *relative risk;* TyG, triglyceride-glucose.
